# Compromised balance control in older people with bilateral medial knee osteoarthritis during level walking

**DOI:** 10.1038/s41598-021-83233-w

**Published:** 2021-02-12

**Authors:** Pei-An Lee, Kuan-Hsien Wu, Hsuan-Yu Lu, Kai-Wen Su, Ting-Ming Wang, Hwa-Chang Liu, Tung-Wu Lu

**Affiliations:** 1 Department of Biomedical Engineering, National Taiwan University, Taipei, Taiwan, ROC; 2 Department of Orthopaedic Surgery, Taiwan Adventist Hospital National Taiwan University Hospital, Taipei, Taiwan, ROC; 3 Department of Orthopaedic Surgery, School of Medicine, National Taiwan University, Taipei, Taiwan, ROC

**Keywords:** Biomedical engineering, Risk factors

## Abstract

About half of the elderly population has knee osteoarthritis (OA), showing altered gait patterns with increased fall risk. The current study aimed to identify the effects of severe bilateral medial knee osteoarthritis on gait balance control, in terms of the inclination angle (IA) of the center of pressure to center of mass vector, and the rate of change of IA (RCIA). Fifteen older adults with severe bilateral medial knee OA and 15 healthy controls walked at their preferred walking speed while the kinematic and forceplate data were measured to calculate IA, RCIA and temporal–spatial parameters. The OA group showed compromised gait balance control, due to a decreased average and range of sagittal RCIA over double-limb support (DLS, p < 0.002) and single-limb support (SLS, p < 0.002), as well as an increased sagittal IA (DLS, p < 0.005). Significantly decreased frontal RCIA averages during DLS, heel-strike and toe-off, and decreased RCIA ranges during SLS and swing (p < 0.027) were also observed. Reducing RCIA during DLS appeared to help reduce the loading rate and pain at the knees, and reduced RCIA at the subsequent SLS. The results indicated an increased risk of loss of balance in the OA group, and may warrant regular monitoring for reduced RCIA during gait to determine fall risk.

## Introduction

Musculoskeletal deteriorations are closely related to balance and gait problems, a major contributor to accidental falls in the older population^1–3^. A musculoskeletal disorder most prevalent in the elderly is knee osteoarthritis (OA), a degenerative disease in which the joint cartilage wears away progressively^4,5^. About 50% of the elderly population above 60 years old have knee OA^4,6,7^, resulting in restricted physical activities with pain and symptoms such as joint swelling and stiffness, muscle weakness, and limited range of motion^8^. These symptoms and the accompanied alterations in joint biomechanics have a direct impact on the gait patterns and balance control^9–12^. Therefore, knowledge of the balance control during gait in people with knee OA may provide useful information for the management of fall risks in this patient population.

Previous studies have reported decreased standing postural stability in older people with unilateral and bilateral knee OA^12–15^. However, clinical observations indicated that falls most frequently occur during activities involving larger displacement of the body’s center of mass (COM), such as walking^16^. Therefore, quantifying standing postural stability alone is not sufficient to describe dynamic balance, and monitoring of the motion of the COM is essential.

Quantifying the motion of the body’s COM relative to the center of pressure (COP) has been used to evaluate the body’s balance control during locomotion^17,18^. A common measure is the COM–COP separation defined by the horizontal distance between the COP and the vertical projection of the COM on the ground. In contrast to static balance during which the downward vertical projection of the COM has to be kept close to the COP, during walking the projected COM can be further away from the COP without loss of balance as long as the COM is controlled at an appropriate velocity relative to the COP^19^. It follows that the COM–COP separation alone cannot indicate the actual control of the COM relative to the COP, as it does not consider the velocity of the COM relative to the COP, nor the influence of the body height or leg length. To overcome the limitations of COM–COP separations, another approach that uses the COM–COP inclination angle (IA) formed by the COM–COP vector and the vertical, and the rate of change of IA (RCIA) has been proposed. The IA combines both the information of COM–COP separation and the height of the COM, and the RCIA integrates the information of the velocity of the COM relative to the COP. The IA and RCIA together provide a more complete description of the COM–COP control, and better enable comparisons between people of different statures^17,20,21^. Generally, interpretation of the IA and RCIA data of a patient group should be made with reference to those of healthy controls in order to identify any particular balance control strategies adopted. For example, an increased IA may suggest a poor balance control, but if one is able to generate an appropriate RCIA either by changing the velocity of the COM or that of the COP or both, the combined changes are considered a successful balance control strategy.

The study of balance control strategies during dynamic activities in terms of IA and RCIA has been reported in various populations^18,20,22^. However, previous studies on patients with knee OA have been limited, and have focused mainly on the COM and IA without considering the velocities of the COM relative to the COP^23,24^. Mandeville et al.^23^ studied the balance control in patients before and after unilateral total knee replacement, examining the maximum COM–COP inclination angles without considering data at gait events and during phases, nor the velocities of the COM relative to the COP. These studies have limited the correct interpretation of the COM–COP data of the knee OA patient group. To the best knowledge of the authors, no study has investigated the effects of knee OA on the balance control in terms of IA and RCIA of the COM motions relative to the COP during level walking.

The purpose of the current study was to identify the effects of bilateral medial knee OA on whole body balance control in older people during level walking, in terms of IA and RCIA. It was hypothesized that older people with severe medial OA would walk with altered, more conservative COM–COP control with increased IA but decreased RCIA when compared to healthy controls.

## Methods

All experiments of the current study were conducted under the approval of Taiwan Adventist Hospital Institutional Review Board (IRB No. 106-E-15). All the experiments and procedures conformed to the Ethical Principles for Medical Research Involving Human Subjects (World Medical Association Declaration of Helsinki). Fifteen older patients with bilateral medial knee OA (OA group), and 15 healthy controls (Control group) matching with the OA group for sex, age and BMI participated in the current study with written informed consent (Table 1). All the patients were determined radiographically to have severe bilateral medial knee OA (KL grade 4) by a senior consultant orthopaedic surgeon with more than 30 years of experience (HCL) using Kellgren and Lawrence criteria^25^ (Table 1). They were also assessed via the Western Ontario and McMaster Universities Osteoarthritis Index (WOMAC) questionnaire. Participants were excluded from the study if they had received an intra-articular corticosteroid injection in the previous two months, or if they had other neuromusculoskeletal diseases or neurological pathology that might affect gait. An a priori power analysis for a two-group independent sample *t* test for the comparison of IA and RCIA between older adults with knee OA and healthy controls based on pilot results using GPOWER^26^ determined that a projected sample size of five subjects for each group would be needed with a power of 0.8 and a large effect size (Cohen's d = 0.8) at a significance level of 0.05. Thus, 15 subjects for each group were more than adequate for the main objectives of the current study.

**Table 1:** Means (standard deviations) of the demographic characteristic of subjects in the knee osteoarthritis group (OA, n = 15) and healthy controls (Control, n = 15).

	OA	Control	p value
Female/male	12/3	12/3	–
Age (years)	66.8 (7.2)	66.5 (7.4)	0.903
Body height (cm)	155.7 (6.6)	156.1 (5.7)	0.888
Body mass (kg)	67.3 (14.7)	61.9 (10.7)	0.279
BMI	27.5 (4.4)	25.4 (4.2)	0.189
Tibiofemoral angle (degrees)	− 3.4 (2.9)	–	–

*OA significantly different from Control.

In a gait laboratory, each subject walked at a self-selected, comfortable pace on a 10-m walkway. The motions of their body segments were tracked by 39 infrared retro-reflective markers placed on specific anatomical landmarks, namely ASISs, PSISs, greater trochanters, mid-thighs, medial and lateral epicondyles, heads of fibulae, tibial tuberosities, medial and lateral malleoli, navicular tuberosities, fifth metatarsal bases, big toes and heels, and mandibular condylar processes, acromion processes, C7, medial and lateral humeral epicondyles, and ulnar styloids^21,27,28^. Three-dimensional (3D) trajectories of the markers were measured using an 8-camera motion analysis system (Vicon MX T-40, UK) at 120 Hz while the ground reaction forces (GRF) were measured simultaneously at 1080 Hz using three forceplates (50.8 cm × 46.2 cm, OR-6-7-1000, AMTI, USA). The motion analysis system has been shown to have a high precision and accuracy with a mean absolute error of 0.15 mm and a variability lower than 0.025 mm^29^, which was considered appropriate for the purpose of the current study. The subjects were allowed to walk on the walkway several times before data collection. Data of a total of six trials, each containing a complete gait cycle for each lower limb, were obtained for each subject.

**Figure 1:**
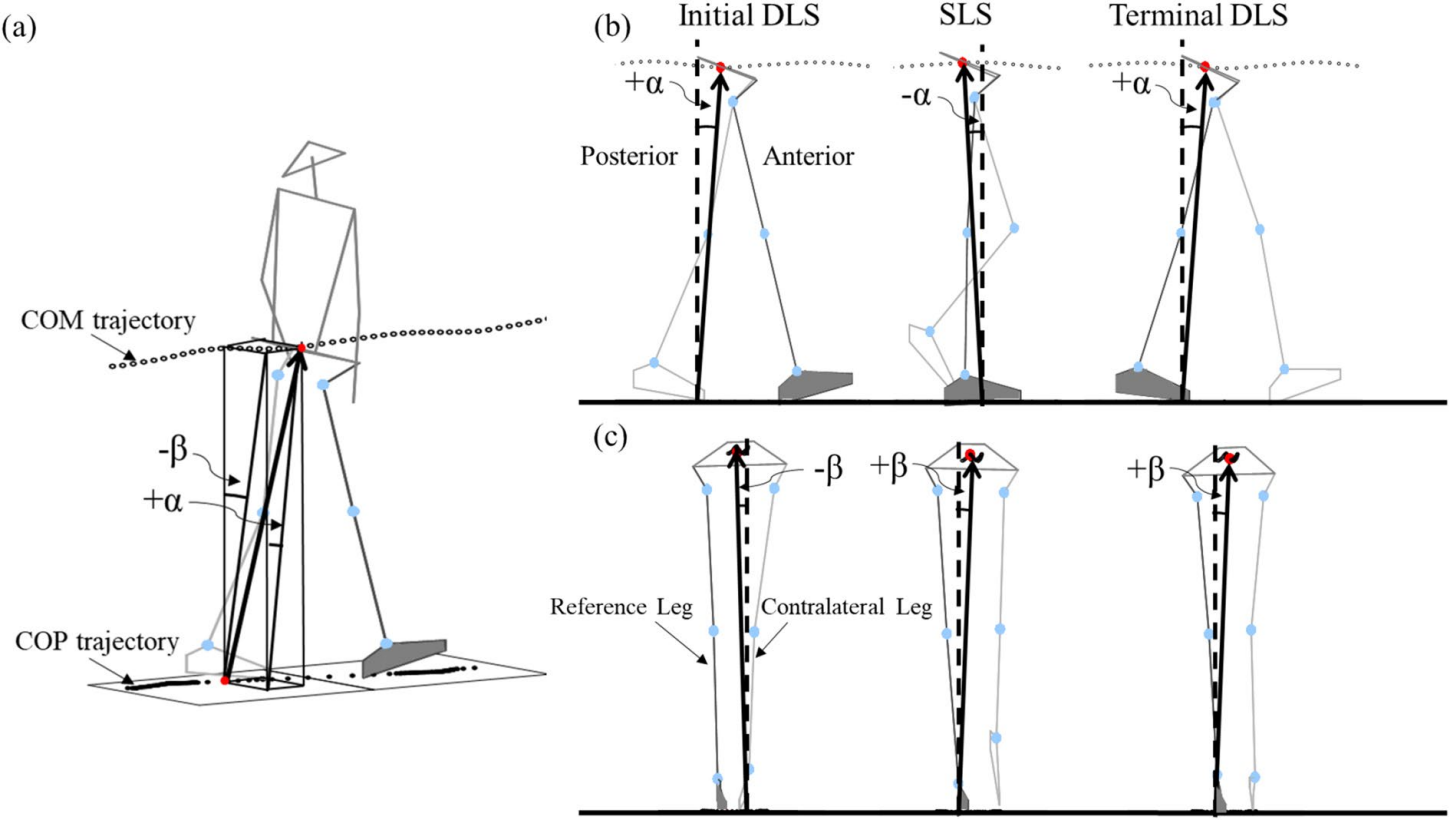
(**a**) Stick figure of the typical subject during level walking showing three-dimensional trajectories of the COM and COP, and the COM–COP vector forming sagittal inclination angles (α) and frontal inclination angles (β) with the vertical. The COM–COP vector and the inclination angles in the sagittal (**b**) and frontal (**c**) planes during initial and terminal double-limb support (DLS), and single-limb support (SLS) are also shown. The reference limb is shown in darker grey.

From the measured marker and GRF data, the mass and position of the COM for each of the body segments were obtained using an optimization-based method^28^, and the body’s COM was calculated as the mass-weighted sum of the COMs of all body segments using a 13-body-segment model. This approach has been shown to have reduced errors in the calculated COM motion when compared to traditional prediction methods^28^. The COP position of the whole body was calculated using the forceplate data. The IA of the COM–COP vector in the sagittal and frontal planes were then calculated as follows:

1 $$\vec{t}=\left(\vec{Z}\times \frac{{\vec{P}}_{COM-COP}}{\left|{\vec{P}}_{COM-COP}\right|}\right)$$

2 $$\mathrm{Sagittal}\,\mathrm{IA}={\mathrm{sin}}^{-1}\left({t}_{Y}\right)$$

3 $$\mathrm{Frontal}\,\mathrm{IA}=\left\{\begin{array}{c}{-\mathrm{sin}}^{-1}\left({t}_{X}\right), for\,\,the\,\,right\,\,limb\\ {\mathrm{sin}}^{-1}\left({t}_{X}\right), for\,\,the\,\,left\,\,limb\end{array}\right.$$

where ${\vec{P}}_{COM-COP}$ was the vector pointing from the COP to the COM, $\vec{Z}$ was the vertical unit vector, and $\vec{X}$ was the unit vector pointing in the direction of progression (Fig. 1). The RCIA were calculated by smoothing and differentiating the IA trajectories using the GCVSPL method^30^. A positive sagittal and frontal IA indicate that the COM is anterior to and away from the COP towards the contralateral limb, respectively. The greater the IA, the greater the horizontal projection of the COM deviates from the COP, and the greater the effort needed to maintain or reduce the deviation unless accompanied by an appropriate RCIA, corresponding to the position and velocity control of the COM as described by Pai and Patton^31^. All the data analyses and graphics generation in the current study were performed using programs in Matlab (R2017b, MathWorks, USA) developed in-house.

For statistical analysis, the values of the IA and RCIA in the sagittal plane and frontal plane at heel-strike and toe-off of both limbs were obtained for each trial and each subject. The ranges of IA and RCIA during the gait cycle, and time-averaged IA over the phases of initial and terminal double-limb support (DLS), single-limb support (SLS) and swing were also obtained. For each of the above-mentioned variables, data from the six trials were averaged. Each of the variables was first tested for normality using a Shapiro–Wilk test. For variables of normal distribution, the between-group differences were tested using independent *t* tests. A significance level of α = 0.05 was set for all tests. All statistical analyses were performed using SPSS version 20 (SPSS Inc., Chicago, IL, USA).

## Results

The average WOMAC subscale values for the OA group were 43 (SD 15.8) for pain, 39.2 (SD 16.9) for stiffness, and 38.3 (SD 7.5) for physical function. Compared to Control, the OA group showed significantly decreased walking speed and cadence with significantly increased step width (Table 2). No significant differences were found in the stride length and step length between the groups (Table 2).

**Table 2:** Means (standard deviations) of the spatiotemporal parameters during walking in the knee osteoarthritis group (OA, n = 15) and healthy controls (Control, n = 15).

	OA	Control	p value
Walking speed (mm/s)	707.4 (119.1)	876.8 (127.13)	0.001*
Cadence (steps/min)	88.2 (13.12)	104.9 (12.63)	0.001*
Stride length (mm)	974.8 (45.85)	1002.7 (54.61)	0.141
Step length (mm)	483.9 (29.45)	497.4 (31.12)	0.231
Step width (mm)	93.9 (18.39)	73.7 (28.9)	0.030*

*OA significantly different from Control.

**Figure 2:**
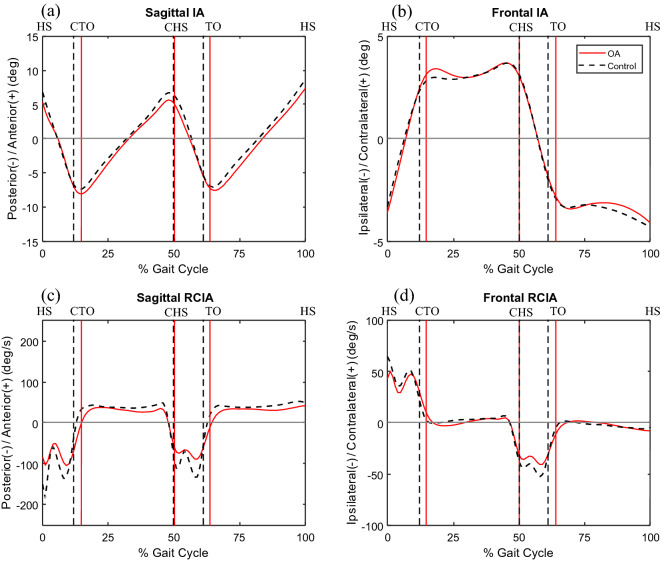
Mean curves of the COM–COP inclination angles (IA) and their rates of change (RCIA) in the sagittal (**a**,**c**) and frontal (**b**,**d**) planes for the knee osteoarthritis group and the control group during level walking. Gait events, namely heel-strike (HS), contralateral toe-off (CTO), contralateral heel-strike (CHS) and toe-off (TO), are indicated by vertical lines. Positive sagittal and frontal IA indicate COM positions that are anterior and contralateral to the COP, respectively. Positive sagittal and frontal RCIA indicate rates of anterior changes and contralateral changes in the corresponding IA, respectively.

**Figure 3:**
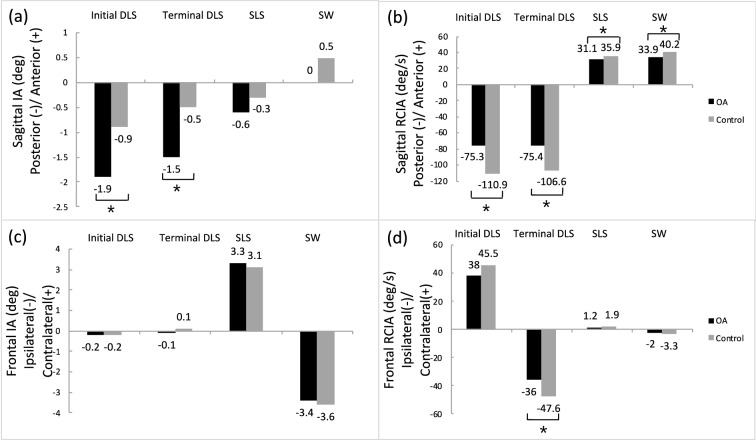
Mean values of time-averaged COM–COP inclination angles (IA) and their rates of change (RCIA) over phases of initial and terminal double-limb support (DLS), single-limb support (SLS) and swing during level walking in the sagittal (**a**,**b**) and frontal (**c**,**d**) planes for the knee osteoarthritis group and the control group. Positive sagittal and frontal IA indicate COM positions that are anterior and contralateral to the COP, respectively. Positive sagittal and frontal RCIA indicate rates of anterior changes and contralateral changes in the corresponding IA, respectively.

Both Control and the OA group showed similar patterns in the IA curves but quite different RCIA curves between OA and Control groups, especially during DLS (Fig. 2). In the sagittal plane, compared to Control, the OA group showed significantly decreased IA at heel-strike but increased average IA during DLS (Fig. 3). The OA group also showed significantly decreased RCIA at heel-strike and toe-off, and during DLS, as well as significantly decreased average and range of RCIA during SLS and swing (Table 3; Fig. 3). In the frontal plane, compared to Control, the OA group showed significantly decreased RCIA at heel-strike and toe-off, and average RCIA during terminal DLS (Fig. 3). They also showed significantly decreased range of RCIA during SLS and swing (Table 4).

**Table 3:** Means (standard deviations) of the sagittal inclination angles (IA) and the rate of change of IA (RCIA) at heel-strike (HS), contralateral toe-off (CTO), contralateral heel-strike (CHS) and toe-off (TO), as well as average values and ranges of IAs during single-limb support (SLS), swing (SW), initial double-limb support (DLSi) and terminal double-limb support (DLSt).

	Sagittal IA (deg)			Sagittal RCIA (deg/s)		
	OA	Control	p value	OA	Control	p value
HS	6.6 (0.9)	7.4 (1.2)	0.028*	− 41.1 (41.4)	− 133.0 (65.6)	0.000*
CTO	− 8.3 (1.1)	− 8.0 (1.0)	0.464	− 1.9 (14.3)	− 38.9 (46.7)	0.010*
CHS	6.9 (0.8)	7.7 (1.3)	0.040*	− 60.8 (38.4)	− 119.1 (53.7)	0.002*
TO	− 8.2 (0.8)	− 7.7 (0.8)	0.100	1.2 (13.6)	− 40.5 (29.0)	0.000*
**Range**						
DLSi	15.4 (1.6)	15.6 (1.7)	0.739	246.0 (76.5)	266.5 (62.8)	0.429
DLSt	15.2 (1.2)	15.4 (1.3)	0.691	251.3 (66.1)	243.9 (39.5)	0.713
SLS	15.5 (1.4)	16.4 (1.1)	0.070	126.4 (35.7)	169.6 (51.3)	0.000*
SW	17.1 (1.7)	18.0 (1.5)	0.116	48.4 (17.5)	101.2 (31.2)	0.000*

P-values for comparisons between knee OA and Control using independent *t* test are also shown. All significance levels were set at α = 0.05.

*OA significantly different from Control.

**Table 4:** Means (standard deviations) of the frontal inclination angles (IA) and the rate of change of IA (RCIA) at heel-strike (HS), contralateral toe-off (CTO), contralateral heel-strike (CHS) and toe-off (TO), as well as average values and ranges of IAs during single-limb support (SLS), swing (SW), initial double-limb support (DLSi) and terminal double-limb support (DLSt).

	Frontal IA (deg)			Frontal RCIA (deg/s)		
	OA	Control	p value	OA	Control	p value
HS	− 4.2 (1.0)	− 3.5 (1.1)	0.092	26.9 (19.8)	50.7 (20.8)	0.003*
CTO	3.3 (0.7)	2.8 (0.7)	0.058	12.0 (6.4)	23.7 (12.6)	0.003*
CHS	3.9 (0.9)	3.7 (0.9)	0.452	− 31.9 (18.5)	− 51.4 (22.4)	0.015*
TO	− 3.4 (0.8)	− 3.2 (0.8)	0.404	− 9.7 (6.8)	− 26.6 (13.3)	0.000*
**Range**						
DLSi	7.1 (1.5)	6.3 (1.6)	0.157	96.1 (36.7)	82.6 (26.3)	0.257
DLSt	7.5 (1.8)	6.8 (1.4)	0.234	93.5 (34.1)	80.5 (24.1)	0.238
SLS	1.3 (0.4)	1.3 (0.6)	0.820	50.0 (20.4)	76.5 (30.0)	0.009*
SW	1.6 (0.8)	1.7 (1.0)	0.842	20.3 (8.4)	33.6 (15.4)	0.006*

p values for comparisons between knee OA and Control using independent *t* test are also shown. All significance levels were set at α  = 0.05.

*OA significantly different from Control.

## Discussion

The current study aimed to identify the effects of bilateral medial compartment knee OA on the whole-body balance control during level walking, in terms of COM–COP IA and RCIA. The OA group was found to walk with an altered COM–COP control throughout the gait cycle, when compared to healthy controls. In the sagittal plane, the patients showed increased IA during DLS but decreased RCIA throughout the gait cycle, while in the frontal plane the IA was not altered but the RCIA was decreased during DLS. These results suggest that bilateral severe medial compartment knee OA compromised the COM–COP control in older adults during gait, which may be related to the increased risk of falling in this population as reported in the literature.

Patients with knee OA in the current study showed increased pain and stiffness, as well as decreased mobility during activities of daily living as indicated by the WOMAC results, in agreement with the literature^23^. These symptoms appeared to contribute to the compromised performance of gait in these patients. For example, decreased gait speed and cadence, and increases in step width may be related to joint stiffness, limited mobility and/or attempts to minimize pain. These gait deviations also contributed directly to the changes in the control of the COM relative to the COP.

During DLS, the OA group walked with the COP moving towards the COM at reduced speed while the COM was controlled within a relatively small range, with similar or increased IA but with significantly decreased RCIA. This indicates that the OA group tended to keep the body weight longer on the trailing limb while transferring the body weight to the leading limb, helpful for reducing the loading rate and pain at the knee joint of the leading limb. It is known that an increased GRF loading rate is associated with increased pain^32^. A plausible reason for this is that damaged cartilage can result in painful abrasive contact between bones^33^ and subsequent difficulties in both shock absorption and the structural support of the body weight. For the current patients with bilateral knee OA, both limbs took turns to serve as the leading weight-accepting limb. Therefore, reducing RCIA during DLS appeared to be helpful for reducing the loading rate and pain at both knees during weight acceptance. However, altered body weight transfer during DLS in the sagittal plane with increased IA magnitude but without an RCIA large enough to maintain the dynamic stability of the COM (Tables 3, 4) may indicate an increased risk of loss of balance^31^. A brief loss of balance can lead to a devastating fall event for older people with knee OA as they may not have to ability to recover balance. On the other hand, the slower weight transfer and reduced forward propulsion during DLS seemed also to lead to reduced RCIA at the subsequent SLS, which further affected the dynamic stability of single-limb support.

During SLS, the patients with knee OA also showed a compromised COM–COP control as found during DLS, primarily in the sagittal plane with significantly decreased RCIA and range of RCIA. During SLS, the base of support is very small and the control of the COM over this small base of support is quite challenging. During this phase, the COP was controlled within a relatively small range while the body COM travelled beyond the supporting foot from a trailing position to a leading position, similar to an inverted pendulum. Reduced RCIA may indicate reduced COM velocity and angular velocity of the inverted pendulum (Tables 3, 4), which reduced the dynamic stability of the system with an increased risk of loss of balance during SLS^31^. In the frontal plane, the COM had a relatively small range of motion during the progression of the body over the stationary foot, with unaltered IA and RCIA. The current results suggest that the patients with knee OA were able to maintain unaltered IA and RCIA with decreased range of RCIA in the frontal plane, even though the projected COM was away from the COP most of the time. Thus, control of the stability may be more difficult to maintain than during DLS.

The current study was the first attempt to identify the effects of severe bilateral medial knee OA on the control of the body’s COM motion relative to the COP during level walking. Generally, the patients with knee OA showed a compromised balance control pattern during walking, especially in the control of the rate of change of the COM to COP motion in both sagittal and frontal planes during DLS, and in the sagittal plane during SLS. While these alterations appeared to be helpful for reducing the loading rate and pain at the knee joint of the leading limb, they may also contribute to an increased difficulty in maintaining dynamic stability of COM–COP motions during walking. Therefore, monitoring for signs of increased IA, reduced RCIA and/or reduced range of RCIA during weight transfer in such patient groups is suggested for identifying an increased risk of falling, especially for those with severe medial knee OA. It is noted that the current results are limited to those with severe knee osteoarthritis. For patients with mild to moderate knee OA, further study will be needed. Another limitation is the unequal number of male and female subjects in both the OA and Control groups. While the female/male distribution of the subjects in the subject groups is a reflection of the actual situation that women have a higher prevalence of knee OA than men^34^, further study would be needed to test whether the current results would be affected by the sex distribution within the group. Further study on the muscle control patterns associated with the balance control strategies in the subject groups using electromyography will be useful for more insight into the changes of neuromuscular control following knee OA. Further studies are also needed to identify how total knee arthroplasty might affect the COM–COP control during walking.

## Conclusions

The patients with severe bilateral medial knee OA showed compromised balance control with altered COM relative to COP motion when compared to healthy controls during level walking, especially reduced RCIA in the sagittal plane throughout the gait cycle, and in the frontal plane during DLS. Reducing RCIA during DLS appeared to be helpful for reducing the loading rate and pain at both knees during weight acceptance, but seemed also to lead to reduced RCIA at the subsequent SLS, further affecting the dynamic stability of SLS. Altered COM relative to COP control without an RCIA large enough to maintain the body’s dynamic stability may indicate an increased risk of loss of balance. Monitoring for signs of reduced RCIA in such patient groups is suggested for identifying their increased risk of falling, especially for those with severe medial knee OA.
